# Tailored Crosslinking Process and Protective Efficiency of Epoxy Coatings Containing Glycidyl-POSS

**DOI:** 10.3390/polym12030591

**Published:** 2020-03-05

**Authors:** Mirjana Rodošek, Mohor Mihelčič, Marija Čolović, Ervin Šest, Matic Šobak, Ivan Jerman, Angelja K. Surca

**Affiliations:** National Institute of Chemistry, Hajdrihova 19, 1000 Ljubljana, Slovenia; mirjana.rodosek@gmail.com (M.R.); mohor.mihelcic@fs.uni-lj.si (M.M.); ervin.sest@gmail.com (E.Š.); matic.sobak@ki.si (M.Š.)

**Keywords:** polymers, octaglycidyl-POSS, DGEBA, dicyandiamide, accelerators, corrosion, protective coatings, infrared spectroscopy, rheology

## Abstract

Versatile product protective coatings that deliver faster drying times and shorter minimum overcoat intervals that enable curing at faster line speeds and though lower energy consumption are often desired by coating manufacturers. Product protective coatings, based on silsesquioxane-modified diglycidyl ether of bisphenol-A (DGEBA) epoxy resin, are prepared through a glycidyl ring-opening polymerization using dicyandiamide (DICY) as a curing agent. As silsesquioxane modifier serves the octaglycidyl-polyhedral oligomeric silsesquioxane (GlyPOSS). To decrease the operational temperature of the curing processes, three different accelerators for crosslinking are tested, i.e., *N,N*-benzyl dimethylamine, 2-methylimidazole, and commercial Curezol 2MZ-A. Differential scanning calorimetry, temperature-dependent FT-IR spectroscopy, and rheology allow differentiation among accelerators’ effectiveness according to their structure. The former only contributed to epoxy ring-opening, while the latter two, besides participate in crosslinking. The surface roughness of the protective coatings on aluminum alloy substrate decreases when the accelerators are applied. The scanning electron microscopy (SEM) confirms that coatings with accelerators are more homogeneous. The protective efficiency is tested with a potentiodynamic polarization technique in 0.5 M NaCl electrolyte. All coatings containing GlyPOSS, either without or with accelerators, reveal superior protective efficiency compared to neat DGEBA/DICY coating.

## 1. Introduction

Typically, alloys corrode merely from exposure to moisture and pollutants in the air atmosphere. According to NACE International (National Association of Corrosion Engineers), the global cost of corrosion is estimated to €2.2 billion, which is equivalent to 3.4% of global gross domestic product (GDP) (<€640 billion or 3.8% GDP in Europe) [1]. Just by available proper corrosion control practices, it is estimated that savings of between 15–35% of the cost of corrosion can be realized (globally €340–800 billion per year) [1]. Commonly used anticorrosion approaches to slow down the corrosion rate are cathodic protection with sacrificial anodes, deposition of protective coatings [2], the addition of inhibitors directly into corrosive environments, or in the structure of protective coatings [3]. Protective coatings are one of the most prospective and widespread methods for the corrosion prevention of metals, which makes it one of the main critical technologies underpinning the competitiveness of the European industry. The deposited coatings isolate the metal surface from the atmosphere or any other corrosive media.

**Figure 1:**
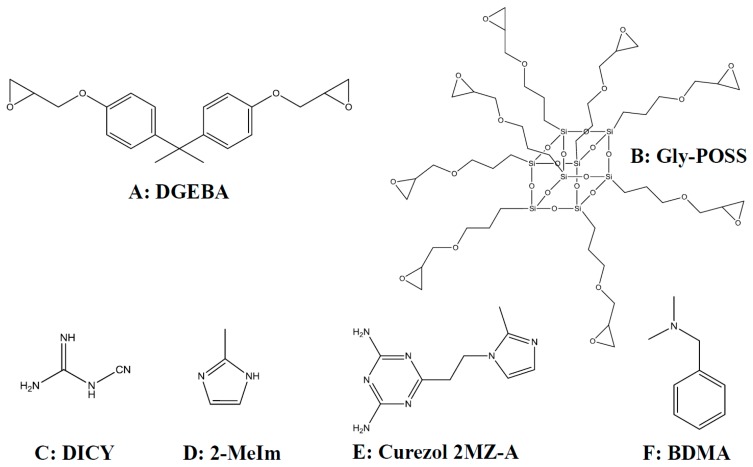
Structures of the precursors: (**A**) diglycidyl ether of bisphenol-A (DGEBA), (**B**) octaglycidyl-POSS (GlyPOSS), (**C**) dicyandiamide (DICY), (**D**) 2-methylimidazole (2-MeIm), (**E**) 2,4-diamino-6-[2’-methylimidazolyl-(1’)]-ethyl-s-triazine (Curezol) and (**F**) *N,N*-benzyl dimethylamine (BDMA).

As a corrosion barrier, the epoxy coatings [4] have been a subject of research and commercial applications for a long time [5]. The well-established routes of their preparation use curing of epoxy-precursors with amino groups-containing compounds; for example, a reaction of diglycidyl ether of bisphenol-A (DGEBA) with dicyandiamide (DICY) curing agent (Figure 1). Despite that, epoxy-based coatings continued to remain a promising topic of investigations through the formation of various composite materials [6,7,8] and advanced metal/polymer laminates [9,10]. For instance, as a challenging issue has remained the development of nanocomposite polymers, in which nanosized reinforcement is applied to obtain improved performance of such protective coatings. Most commonly, nano reinforcement compounds of <100 nm size have been added to the polymeric matrix, and the uniform dispersion is the crucial key for achieving the desired properties. Nanoparticles are commonly introduced as fillers [11] but can also be bound directly into the polymeric matrix as pendant groups.

Nanoparticles that can, for example, be bound in the epoxy networks are polyhedral oligomeric silsesquioxanes (POSS) [12]. They belong to a group of organic-inorganic hybrids [13]. Specifically, the core is composed of an inorganic silsesquioxane, while different organic pendant groups can be attached to each of the eight corners of the T_8_ cage. Homoleptic POSS nanoparticles have the same organic groups in their shells, while organic groups in heteroleptic POSS can differ. Consequently, POSS can incorporate in different modes into novel materials. (i) When no reactive organic group is present in the shell, POSS simply behaves as a nanofiller. (ii) On the other hand, the presence of a suitable reactive organic group can lead to the bounding of POSS into the epoxy network. POSS can enter the epoxy curing reaction either as: (ii-a) glycidoxy-group containing precursor or (ii-b) an amine-POSS hardener compound. (ii-a) The examples of the former approach are epoxy networks based on DGEBA that contained different fractions of monoglycidyl-heptaisobutyl-POSS [14,15,16,17] and were cured with different hardeners. Strachota et al. [18], on the other hand, used POSS with 1, 2, 4, or 8 glycidyl groups while remaining groups to T_8_ cage being either phenyl, isooctyl, or cyclopentyl. (ii-b) The latter approach was demonstrated by partial exchange of hardener with either monoamino-functionalized POSS [17,19,20,21] or octaamino-functionalised POSS [22]. (iii) Nevertheless, the open-cube trisilanol-heptaphenyl-POSS was studied as a promotor of the curing reaction between glycidyl and amino groups, i.e., the influence of silanol groups on epoxy curing kinetics [23].

By incorporation of POSS nanoparticles into polymer matrices, the composite materials with superior functional properties can be obtained. For example, a combination of epoxy and various glycidoxy-POSS nanoparticles have been studied from mechanical [14] and viscoelastic [18] perspective. It was found that monoglycidoxy-POSS does not contribute to the deformation process of the network while enhancing the thermal properties [14]. The thermal stability of epoxy/glycidyl-POSS materials is improved, but higher loadings tend to decrease this beneficial influence [18]. The behavior of DGEBA with monoglycidoxy-heptaisobutyl-POSS cured with short aromatic amines was studied from the kinetic perspective by differential scanning calorimetry (DSC) [16]. In a similar material, local thermal analysis (LTA) and DSC gave evidence of amorphous POSS-rich domains which can eventually arise from phase separation [15]. As the main reason for that, the incompatibility of the isobutyl groups of monoglycidoxy-heptaisobutyl-POSS and the aromatic epoxy-amine network was suggested. Anyhow, when octaglycidoxy-functionalized POSS (Figure 1B) was introduced into the DGEBA-based nanocomposite system, it was found to accelerate the rate of opening of glycidyl epoxy rings of DGEBA [24].

POSS molecules also have a robust resistance to environmental degradation factors, such as moisture, oxidation, corrosion, and UV radiation. It is therefore not surprising that various POSS nanoparticles have already been tested as an additive in polymeric protective coatings for alloys, for example, aminopropyl-heptaisooctyl-POSS in epoxy coatings [20,21]. However, although epoxy coatings are used worldwide as corrosion protective coatings [7], we haven’t found any research report on the corrosion topic where the glycidyl-POSS molecules are added to improve the protective efficiency of epoxy coatings. Much more obvious are reports on the addition of different silanes [8] or the application of silane primers [5]. Since the addition of monoglycidyl-heptaisobutyl-POSS nanoparticles can cause phase separation, as hydrophobic isobutyl groups are not compatible with the epoxy network [15], the much easier is to incorporate homoleptic octaglycidyl-POSS (abbreviated GlyPOSS in Figure 1) molecules in the epoxy matrix [24]. Such molecule can be represented by formula (R–SiO_1.5_)n (n = 8, R = –(CH_2_)_3_–O–CH_2_–[C_2_H_3_O]). Under this premise, it is expected that the developed coatings shall have lower curing and glass transition temperature (*T*_g_), lower roughness, dense structure, and consequently, also the outstanding protective properties.

To achieve the dense coating structure and other above-listed properties, special attention has to be given to the selection of the amine hardener. In reports on epoxy composites with glycidyl-POSS nanoparticles as hardeners, different aromatic amines [15,16] and polyetheramines (Jeffamines) [14,18] were studied. However, the small aliphatic molecule of dicyandiamide (DICY in Figure 1C) also suggests that dense crosslinking of the epoxy matrix is possible [7].

In order to decrease the production cost of protective coatings, the lowering of curing temperature, is desired. This can be achieved through the acceleration of curing reactions, i.e., the addition of suitable accelerators. The lowering of the activation energy for glycidoxy ring opening can be achieved by the presence of proton donors, for example, alcohols or hydroxyl groups emerging from previous reactions [25]. Further lowering of the ring-opening temperature is achieved by the addition of tertiary amines [25]. Different tertiary amines have been tested for DGEBA-based systems, ranging from benzyl dimethylamine (BDMA in Figure 1F) to various imidazolium-based structures [26,27]. The results confirmed the influence of tertiary amine accelerators on the curing dynamics and resulting materials. This suggests that specific studies should include different accelerators.

Herein, we report on a successful preparation of composite epoxy protective coatings for aluminum alloy AA 2024. Part of DGEBA precursor is exchanged by GlyPOSS to decrease the influence of bisphenol-A on public health, to decrease the production costs, and to achieve better protective efficiency of coatings. Three different amine groups-containing accelerators, i.e., *N,N*-benzyl dimethylamine (BDMA), 2-methylimidazole (2-MeIm), and commercial accelerator Curezol (Figure 1D–F), are compared regarding the triggering of the curing reaction. They are studied regarding their capacity for the opening of glycidoxy rings but also their eventual contribution to crosslinking. The influence of accelerators on the curing is proved via thermal- and time-dependent FT-IR spectroscopy and rheological examination. Differential scanning calorimetry (DSC) is used to determine the thermal properties of epoxy-octaglycidyl-POSS composites. Morphology of the coatings is checked using atomic force microscopy (AFM) and scanning electron microscopy (SEM). The electrochemical technique, i.e., potentiodynamic polarization, gives a clear answer on improved protective efficiency of the developed epoxy-GlyPOSS coatings.

## 2. Materials and Methods 

### 2.1. Materials

Diglycidyl ether of bisphenol-A (DGEBA) was obtained from ABCR (Karlsruhe, Germany), as well as solvent 2-butanone (ACS, 99%). Commercial octaglycidyl-POSS (abbreviated GlyPOSS) nanoparticles were purchased from Hybrid Plastics (Hattiesburg, MS, USA). Curing agent dicyandiamide (DICY) and accelerators 2-methylimidazole (2-MeIm, 99%) and *N,N*-benzyl dimethylamine (BDMA, 99%) were purchased from Sigma-Aldrich (St. Louis, MO, USA). Commercial accelerator Curezol 2MZ-A (abbreviated Curezol) was obtained from Air Products (Allentown, PA, USA). Dimethyl sulfoxide (DMSO) was purchased from Merck (Darmstadt, Germany). All chemicals were used as supplied.

### 2.2. Preparation of Coatings

**Figure 2:**
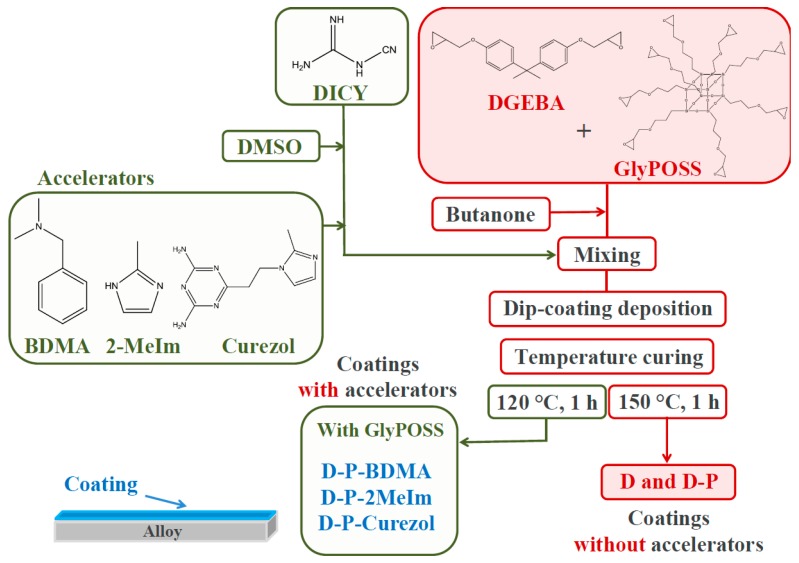
The preparation procedure of the protective coatings.

The preparation procedure of coatings is depicted in Figure 2. DGEBA (1.6 g) and GlyPOSS (0.4 g) were dissolved in butanone (2 g) and stirred for 30 min. For coating without GlyPOSS, only 2 g of DGEBA was dissolved in butanone (2 g). Separately, DICY (0.25 g) was dissolved in DMSO (1 g). Into the latter solution, if appropriate, one of three accelerators was introduced in molar ratio DICY:accelerator = 1:0.1. Both solutions were finally mixed. Before dip-coating deposition on aluminum alloy AA 2024 (Aviometal, Italy) with a pulling velocity of 10 cm/min, mixtures were left to stir 5 min. The substrates (dimensions: 2 × 5 cm^2^) were polished using 3 M Perfect-IT III paste and subsequently sonicated in hexane, acetone, and methanol for 15 min. Specifically, for this research five types of coatings were prepared:

- DGEBA/DICY (D coating) without the accelerator,
- DGEBA/GlyPOSS/DICY (D-P coating) without the accelerator,
- DGEBA/GlyPOSS/DICY/BDMA (D-P-BDMA) coating,
- DGEBA/GlyPOSS/DICY/2-MeIm (D-P-2MeIm) coating,
- DGEBA/GlyPOSS/DICY/Curezol (D-P-Curezol) coating.

In parenthesis, the abbreviations that are depicted on graphs are shown. The curing process was performed at 150 °C for 1 h for coatings without accelerators and at 120 °C for 1 h for coatings with accelerators.

### 2.3. Methods

FT-IR absorbance measurements were made on a Bruker spectrometer, model IFS 66/S (Bruker, Billerica, MA, USA). All samples, i.e., either precursors or formulations, were deposited on silicon wafers. Temperature-dependent FT-IR absorbance measurements, from room temperature to either 120 or 150 °C, were performed in Spectra-Tech heated demountable cell with a controller. When the desired temperature was achieved, the spectrum was recorded. The spectra were gathered every 10 min during measurements at 120 and 150 °C. The resolution was 4 cm^−1^. 

DSC measurements were performed on a Mettler-Toledo DSC-1 (Columbus, OH, USA) calorimeter under a nitrogen atmosphere with a flow rate of 50 mL/min. Samples were sealed in 40 μL alumina crucibles with the lids. The mass of the samples was around 10 mg for all lyophilized mixtures. The analysis was performed with a heating rate of 5 K/min from −30 to 300 °C. 

The rheological behavior of the epoxy resin was observed by nonisothermal dynamic oscillation with a rotational controlled rate rheometer (Physica MCR301, Anton Paar, Graz, Austria), equipped with a parallel geometry (PP-25). The epoxy samples were heated by convection. A solvent trap was used to minimize solvent evaporation, while a temperature-controlled hood was applied to prevent heat dissipation. The measurements were performed under a constant shear strain (10–20%) with a gap of 0.5 mm. Dynamic measurements were performed using a heating rate of 2 °C/min from 23 to 180 °C under a constant flow of dry nitrogen to eliminate any oxidative processes during heating. 

Taylor Hobson Series II profilometer (Leicester, United Kingdom) was used for coatings thickness determination. Coatings were characterized by a scanning electron microscope FE-SEM Zeiss SUPRA 35VP (Zeiss, Oberkochen, Germany). Atomic force microscopy (AFM) images were made on an AFM attachment of WITec alpha 300 confocal Raman spectrometer (Ulm, Germany). The images were recorded on areas of 10 × 10 µm^2^ of the prepared coatings deposited on AA 2024 coupons. The images are presented in the two-dimensional representation without filtering. According to the scale bars alongside the images, the brighter the color, the higher the spot on the surface. Consequently, surface roughness (*SR*) was calculated. Samples were measured at room atmosphere and temperature. 

An Autolab PGSTAT30 potentiostat-galvanostat (Metrohm Autolab, Utrecht, The Netherlands) was used to perform electrochemical measurements. Potentiodynamic polarization measurements were made in a K0235 flat cell (Ametek Scientific Instruments, Oak Ridge, USA) with a built-in Pt grid counter electrode. The cell was filled with a 0.5 M NaCl electrolyte. The coating on AA 2024 was mounted as a working electrode, while Ag/AgCl/KCl_sat_ served as a reference electrode. The coating was held at an open circuit potential for 30 min before the measurement. Then linear sweep voltammetry was swept from 1.0 to 0.0 V using a scan rate of 1 mV/s. The corrosion current density (*j*_corr_) was extrapolated with Tafel slopes from the measured potentiodynamic polarization curves.

## 3. Results

### 3.1. Characteristics of Formulations and Curing Process

**Figure 3:**
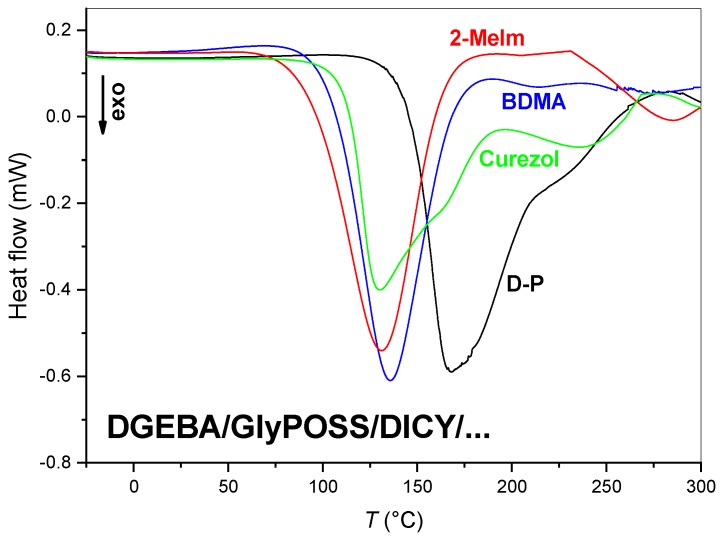
Normalized differential scanning calorimetry (DSC) curves of the D-P-based formulations.

**Figure 4:**
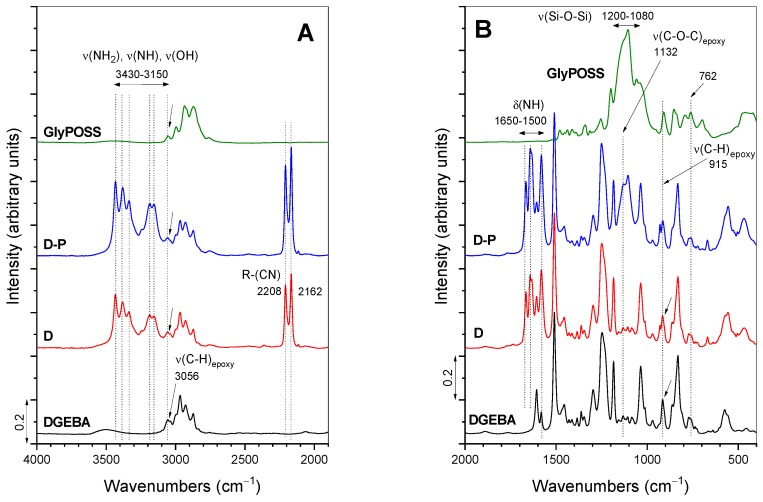
FT-IR absorbance spectra of precursors DGEBA and GlyPOSS, and initial D and D-P formulations in the spectral ranges: (**A**) 4000–1900 cm^−1^ and (**B**) 2000–400 cm^−1^.

**Figure 5:**
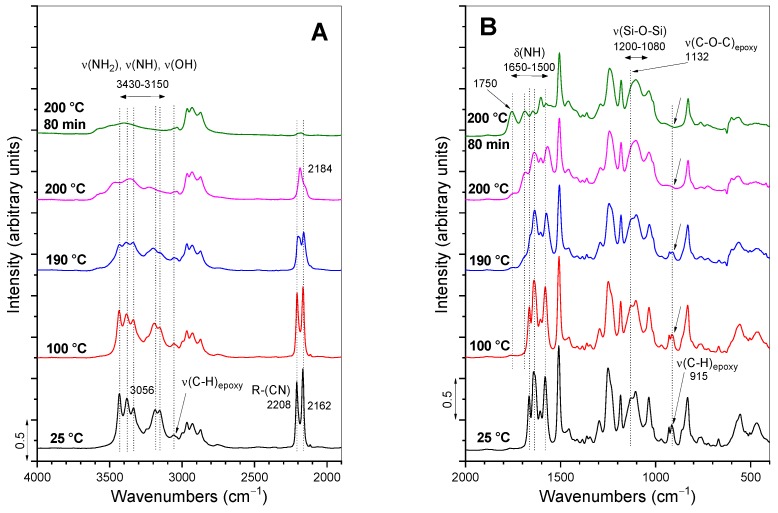
Time-dependent FT-IR absorbance spectra during the thermal curing process of formulation D-P in the spectral ranges: (**A**) 4000–1900 cm^−1^ and (**B**) 2000–400 cm^−1^.

**Figure 6:**
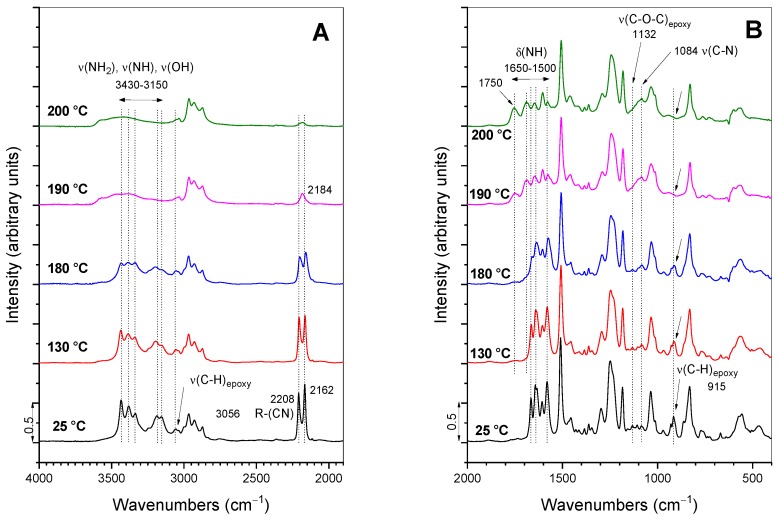
Time-dependent FT-IR absorbance spectra during the thermal curing process of formulation D in the spectral ranges: (**A**) 4000–1900 cm^−1^ and (**B**) 2000–400 cm^−1^.

**Figure 7:**
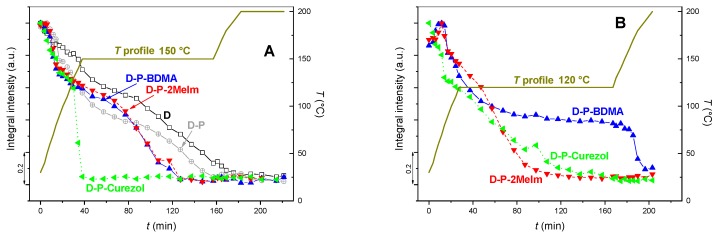
Time-dependent integral intensity changes in the characteristic epoxy band of 915 cm^−1^ with thermal curing. The temperature profile that was followed with time is depicted, as well: (**A**) 150 °C and (**B**) 120 °C.

**Figure 8:**
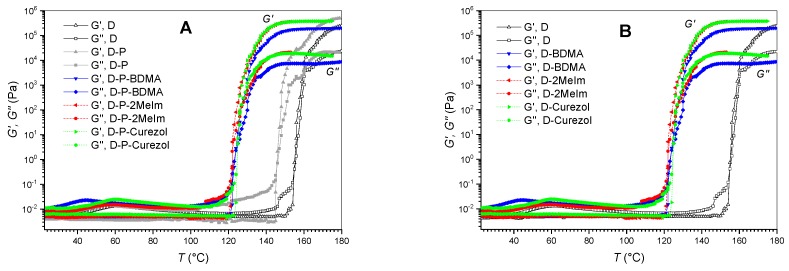
Rheological characterization formulations during thermal curing: (**A**) D-P-based and (**B**) D-based formulations.

Epoxy-based formulations belong to thermosetting materials that cure upon heating. Insight into curing dynamics can be obtained using different analytical techniques. DSC analysis, for example, provides information on glass transition temperature (*T*_g_), start and completion of the curing process and the enthalpy of the curing process (Figure 3). Time-dependent FT-IR absorbance measurements during temperature treatment of coatings (Figure 4, Figure 5 and Figure 6) enables insight into the crosslinking by observation of decreasing intensity of the epoxy mode at 915 cm^−1^ [28]. Rheology (Figure 7 and Figure 8), on the other hand, gives quite a piece of information on changes in consistency during the transformation of the formulation into a crosslinked structure [18,29]. When the obtained results are observed in parallel experiments, designed to approach to the same conditions as much as possible, they can throw light to the curing processes that lead to the formation of highly crosslinked protective coatings. The important point of this study is to follow the influence of accelerators on the curing process. We look forward to connecting their structural properties with the behavior during the curing of formulations.

#### 3.1.1. Thermal Properties

DSC analysis showed that for all curves of D-P-based formulations, the characteristic exothermic peaks appear (Figure 3). The curing of the D-P formulation without accelerator took place in the range of 130–280 °C and the enthalpy of the reaction reached 506 J/g (Table 1). As expected, the addition of any of the three accelerators speeded up the curing. Consequently, the curing maximums were found at approximately 30–40 °C lower temperatures (Table 1). The enthalpy detected for formulations with accelerators was lower for 100–160 J/g, meaning that some reactions already started during the lyophilization preparation procedure. In the case of BDMA and 2-MeIm accelerators, this phenomenon was more prominent as the crosslinking reaction started at lower onset temperatures (*T*_onset_: 2-MeIm < BDMA < Curezol). The shapes of DSC curves of D-P and D-P-Curezol formulations were the same indicating that the behavior of Curezol is similar to DICY. Namely, Curezol acts as an epoxy ring-opening initiator (2-methyl imidazole part of its molecule accelerates and shifts the curing reactions to lower temperature) and as a crosslinking agent (through amino groups on the triazine ring). However, the Curezol accelerator shifted the D-P-Curezol curve to lower onset temperature. As Curezol, also 2-MeIm can act in both roles, i.e., as ring-opener and crosslinking agent. The crosslinking role of imidazoles was confirmed already long ago by NMR studies [30]. In addition, it was proposed that imidazole ring in the polymer matrix positively influences its physical and chemical properties [30].

It is commonly accepted that accelerators in epoxy resins decrease the curing temperature and enhance the curing rate. As described above, DSC curves (Figure 3, Table 1) confirmed that for all three tested accelerators in our D-P system. The addition of any accelerator also led to a change in *T*_g_ value [30,31,32]. However, these changes vary in both directions with regard to the *T*_g_ value of 116 °C obtained for D-P formulation without accelerator (Table 1). Interestingly, the *T*_g_ value increased in case of BDMA and Curezol, but decreased for accelerator 2-MeIm. It is reported for the DGEBA+DICY system that the increasing amount of accelerator 1-ethyl-3-methyl-imidazolium dicyanamide consistently decrease the *T*_peak_ temperature. All described formulations exhibit *T*_g_ above 120 °C and the values increased slightly with the increase in the concentration of the accelerator 1-ethyl-3-methyl-imidazolium dicyanamide. On the other hand, in a similar DGEBA+DICY system with either BDMA or 2-MeIm accelerators showed similar values of *T*_g_ to ours, i.e., 115–116 °C for the 2-MeIm accelerator and 139–147 °C for the BDMA accelerator depending on cure characteristics [32]. More detailed dependency studies of *T*_g_ were performed for the BDMA accelerator [32]. Specifically, the *T*_g_ value is dependent on the concentration of the BDMA accelerator and ratio DICY/BDMA, which was confirmed with the maximal *T*_g_ value achieved in the middle of the tested concentration profile. Moreover, dependency of *T*_g_ on amine/epoxy ratio in systems with BDMA was also demonstrated [32]. It can be concluded though that the *T*_g_ value considerably depends on the systems and should be more detailed studied for each system separately. This will be made in our future work.

Some studies observed the appearance of the second peak in DSC curves, as was noted for accelerator 1-ethyl-3-methyl-imidazolium dicyanamide [31]. Such peaks became more dominant with the increasing amount of the imidazolium salt accelerator. It was suggested that the second peaks can be tentatively attributed to the more pronounced reaction of DICY (since the addition of this accelerator decreased the amount of the unreacted DICY [31]. In addition, this aspect remains for our future concentration-dependent investigation of our system.

#### 3.1.2. Temperature-Dependent FT-IR Spectra

The characteristic bands in the FT-IR absorbance spectra of precursors (DGEBA, GlyPOSS) and formulations D and D-P are evident from Figure 4 and Figure S1, the latter in Supporting Information. The epoxy modes appear in the spectrum of DGEBA at 3056 (ν_s_(C–H)_epoxy ring_), 1132 (ν(C–O–C)_epoxy ether_) and 915 (ν(C–O)_epoxy ring_) cm^−1^ [5,16,28]. The first two bands are of low intensity and in the proximity of other modes. The intensity of the band at 915 cm^−1^ is moderate and can serve as a measure of the extent of the curing reaction, i.e., opening of glycidoxy rings and their crosslinking into polymeric materials [28]. The spectrum of D formulation, in addition to vibrations of DGEBA, shows the stretching of primary and secondary amines (ν(NH_2_), ν(NH)) between 3430 and 3150 cm^−1^ and cyano group at 2208 and 2162 cm^−1^ [33] (Figure 4A). When DGEBA is in part exchanged with GlyPOSS, i.e., for D-P formulation, the spectrum retains the basic characteristics of amino and cyano groups (Figure 4A). As well, the epoxy bonds at 3056 and 915 cm^−1^ remained visible in this spectrum, but the low-intensity epoxy band at 1132 cm^−1^ became overlapped with the broad bands in the spectral region 1200–1080 cm^−1^. They belong to the stretching vibrations of ν(Si–O–Si) originating from the silsesquioxane cage of GlyPOSS. Such intensive ν(Si–O–Si) band was also noted in the spectrum of GlyPOSS/4,4′-(1,3-phenylenediisopropylidene) epoxy composites [24]. Importantly, in that study, the appearance of the glycidoxy groups of GlyPOSS is reported at ~745 cm^−1^ [24]. In our spectrum of GlyPOSS, the nearest band that can be assigned to glycidoxy groups is at 762 cm^−1^ (Figure 4B). However, it overlaps with the glycidoxy groups of DGEBA. Therefore, it only marginally contributes to the intensity increase of the 762 cm^−1^ band in D-P formulation regarding the D formulation. For this reason, it cannot be used to follow the curing of GlyPOSS glycidoxy groups.

The FT-IR absorbance spectra obtained during the curing of D-P formulation with temperature is evident from Figure 5 and, for comparison, for D formulation in Figure 6. The whole spectra are shown in Figures S2 and S3 in Supporting Information. The spectrum recorded at 100 °C for D-P formulation already showed a notable change (Figure 5). Specifically, the reactions between the opened glycidoxy rings of DGEBA, GlyPOSS and primary and secondary amine groups of DICY started to occur. The result was also the formation of hydroxyl groups [5,25], while the opening of glycidoxy rings of DGEBA was indicated through the decrease in the intensity of the above-mentioned epoxy stretching bands at 3056 and 915 cm^−1^. Regarding the low intensity of the 3056 cm^−1^ band, the 915 cm^−1^ band (Figure 5) remains the most appropriate one for the following of this curing reaction through a calculation of its integral intensity [28]. The uncertainties can occur in the final curing stages when the concentration of glycidoxy rings became very low [28]. As the reference band for normalization, 1509 cm^−1^ band of C–C stretching of the aromatic ring was taken.

The same trend, i.e., decrease in the intensity during curing (Figure 5), was observed for the bands associated with amines of DICY in the spectral region 3430–3150 cm^−1^ (ν(NH_2_), ν(NH)) and 1650–1500 cm^−1^ (δ(NH)). According to the literature, cyano–CN groups (2208 and 2162 cm^−1^) can also provide crosslinking at higher temperatures [31]. Such network crosslinking in the formulation is evident from the appearance of the stretching ν(C–N) band at 1084 cm^−1^ at temperatures around 190 °C. Since this band is overlapped by ν(Si–O–Si) bands of silsesquioxane GlyPOSS in Figure 5, its evolution can only be followed in the spectra of D formulation (Figure 6; Figure S3 in Supporting Information). The curing process of D-P formulation was finished after 80 min at 200 °C (Figure 5). The main characteristics of the FT-IR bands with curing were similar, in the case of D formulation (Figure 6). The spectra revealed the decrease in intensity of epoxy, amino and cyano bands at 200 °C. As mentioned above, due to the absence of ν(Si–O–Si) bands, the increase in the intensity of stretching ν(C–N) band can be observed (Figure 6B). 

The spectra of D-P-accelerator formulations revealed similar features than the described D-P one (Figure 5) due to the low concentration of accelerators. Consequently, the time-dependent FT-IR spectra during thermal curing are not shown for the formulations with accelerators. Anyhow, the influence of accelerators on the course of the curing reactions is shown through the presentation of the integral intensity decrease in the 915 cm^−1^ band for either of the formulations (Figure 7). The curing was performed up to either 120 or 150 °C, at which the process was followed for a certain period, before the continuation with the temperature increase. The spectra of formulations without accelerators (D, D-P) were only cured with the 150 °C temperature profile (Figure 7A).

The curing of D and D-P formulations occurred quite gradually (Figure 7A). For D formulation two slopes can be discerned. When GlyPOSS was added to the coating formulation (D-P), the integral intensity of the 915 cm^−1^ band decreased slower during the initial 20 min, but then somewhat more abruptly around 60 °C. After reaching the isothermal temperature of 150 °C, the initial increase in slope is followed by some relaxation and then a steady decrease. The integral intensity approached zero after ~2 h of isothermal treatment for either of formulations, D or D-P. The observed differences in the intensity decrease reflect the composition of both formulations. The incorporation of GlyPOSS molecules into the epoxy matrix of D-P formulation induced the sterical hindrances. Specifically, such molecules are equipped with eight reactive groups extending into all directions. When only DGEBA and DICY are the reactive species in the D formulation (Figure 6; Figure S3 in Supporting Information), the formation of the epoxy matrix demands the approaching of the reactive amine groups to glycidoxy rings. The addition of GlyPOSS with eight glycidoxy rings in the corners of the cube silsesquioxane, however, enabled their bonding without any necessary preceding orientation of the silsesquioxane. Consequently, the crosslinking occurred somewhat faster at certain temperatures, which becomes apparent at 60 °C. After that temperature, the inclinations of the slopes are similar for both formulations, i.e., D and D-P, throughout the whole isothermal treatment.

The addition of accelerators to D-P formulation changed the course of the integral intensity curves (Figure 7A). During the temperature increase up to 150 °C, the integral intensity of the 915 cm^−1^ band decreased quicker compared to that of the neat D-P formulation. Interestingly, the accelerators BDMA and 2-MeIm behaved similarly. Such behavior was expected since at this temperature these two accelerators are characterized as compounds that catalyze the opening of glycidoxy rings [32]. When accelerator Curezol was added, the integral intensity decreased abruptly after reaching 130–140 °C (after 30–40 min in Figure 7A). This is understandable since the Curezol—in addition to the opening of the glycidoxy rings—can also contribute to the crosslinking reactions (post-curing) through primary amino groups at the triazine ring (Figure 1E). 

The inspection of the integral intensity behavior of the 915 cm^−1^ band during a 120 °C temperature profile (Figure 7B) revealed the difference among both accelerators with the predominantly catalytic activity. Initially, during heating to 120 °C (40 min in Figure 7B), the intensity decrease was similar for BDMA and 2-MeIm accelerators. However, during isothermal treatment at 120 °C, the integral intensity remained considerably constant when BDMA was added. It did not continue to decrease until 150 °C (190 min) was reached, showing that BDMA possesses only the ring-opening properties. In contrast, formulation with 2-MeIm showed a considerable decrease in the intensity of the 915 cm^−1^ band already during the isothermal treatment. The described discrepancy pointed out that the 2-MeIm as aromatic amine is an epoxy ring-opener which can be after initiation ingrained in the polymer structure [31]. Curezol remained the quickest in its action that occurred through an opening of glycidoxy rings and crosslinking up to 120 °C, while during isothermal treatment, it approached the intensity behavior of formulation with 2-MeIm. 

#### 3.1.3. Rheological Characteristics

With the same aim, i.e., to investigate the influence of different accelerators on the curing of D-P-based formulations, rheological characterization was made. Figure 8 shows the dynamic storage modulus (*G*′) and loss modulus (*G*″) versus temperature during heating at a heating rate of 2 °C/min. These rheograms confirm that curing processes are time- and temperature-dependent [18,29]. Although the shapes of the curves obtained for elastic and viscous modulus are similar for all formulations, the curing reactions start at considerably different temperatures (i.e., times). However, *G*′ or *G*″ tend to final values of the order of magnitude 3 × 10^5^ Pa (*G*′) and 10^4^ Pa (*G*″) for all formulations, respectively. The evolution of the elastic *G*′ modulus, which is proportional to the rigidity of the cured epoxy, indicate the formation of molecular networks through the formation of chemical bonds (crosslinking) in the either of the investigated epoxy systems [29]. 

The curves of the basic formulation D (Figure 8, Table 2) reveal that up to approximately 154 °C when the viscous modulus *G*″ dominated the elastic *G*′ one, this epoxy system was in a viscous state (*G*″ > *G*′). This gradual increase in the *G*″, viscous modulus shows that the reaction processes have already started in the formulation. The crosslinking, however, occurred at 66 min and the temperature of 155 °C, when *G*′ equals *G*″ (Table 2). Afterward, the gel state occurred, but the values of elastic and viscous moduli continue to increase. The elastic *G*′ portion prevails the viscous *G*″ one thereupon. The final stage of the curing process is the formation of the plateaus for both *G*′ or *G*″, indicating the cured epoxy system. The more significant is the difference between both moduli in the plateau region, the stronger is the cured epoxy system (i.e., more rigid solid matter). 

The processes that are described for the D formulation also occurred in other investigated systems but at a different temperature-scale (Figure 8). In D-P formulation, the crosslinking happened already after 62 min and the temperature of 146 °C (Table 2). Other characteristics of the curing processes remained similar as for D formulation. However, the absolute value of elastic modulus *G*′ reached higher values for the formulation with GlyPOSS compared to neat D formulation. This indicated that more crosslinked and stiff structure formed when GlyPOSS with eight glycidoxy groups steaming from the siloxane cube center was added. 

The addition of any accelerator (BDMA, 2MeIm, Curezol) to D-P formulation contributed to even faster crosslinking (~50 min) and at approximately 22 °C lower temperatures (Figure 8A, Table 2). Differences among accelerators were small. Interestingly, the differences between plateau *G*′ and *G*″ values were similar for all three accelerators, but when BDMA was added, both plateau were shifted to somewhat lower values. This indicates that the extent of the crosslinking of the internal matrix was the lowest in the case of BDMA accelerator (as it did not take part in crosslinking), which is in the correlation with FT-IR results (Figure 7B) as more or less sufficient crosslinking was achieved at 120 °C. When 2-MeIm or Curezol were used, the achieved moduli *G*′ and *G*″ were similar to the ones obtained by D-P formulation, resulting in a similar crosslinking. These two accelerators could also collaborate in crosslinking, they are not only epoxy ring-openers. BDMA only acts as the epoxy ring-opener. 

As described above, rheology was used to check the influence of accelerators on the D-P system (Figure 8A, Table 2). Besides, we evaluated their influence on sole D formulation (Figure 8B, Table 2). At first glance, the temperature of crosslinking is quite similar for either of the D-accelerator formulations. However, if an average value is calculated out of temperatures of crosslinking for formulations with accelerators, the average temperature obtained for D-accelerator formulation is about 1 °C lower (Table 2). This implements that accelerators can exert a slightly larger influence on D formulation compared to D-P formulation. Such an effect can be understandable since the introduction of GlyPOSS brings about some sterical hindrances and tensions during the packaging of the compounds into the crosslinked solid material.

**Figure 9:**
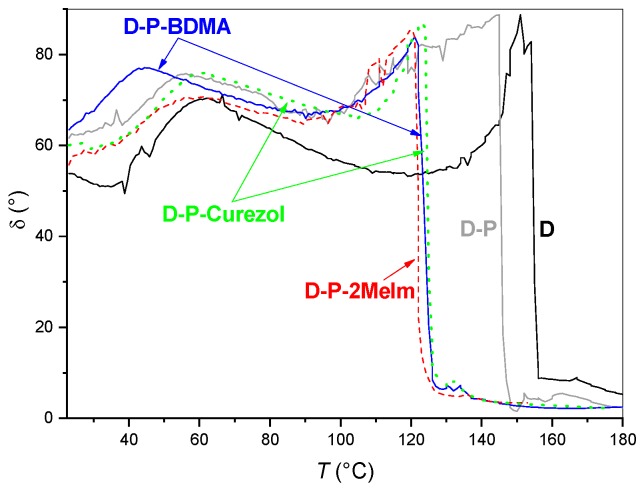
Phase angles of D, D-P and D-P-accelerator formulations during curing.

The typical characteristics of the crosslinking processes in the investigated epoxy formulations can also be viewed through the behavior of phase angle (Figure 9). This parameter is defined as a ratio between the lost and the stored deformation energy, which means the ratio between the viscous and the elastic contribution to the viscoelastic behavior. Namely, the proper balance between the viscous and the elastic contribution in the viscoelastic formulation before the deposition of the coatings is of particular importance when our aim is the deposition of the protective coatings with excellent barrier properties [18,29].

Specifically, the phase angle *δ* shows the time delay between the determined sinusoidal parameters and the measured characteristics *G*′ and *G*″. These differences occur due to the viscosity changes of the measured formulations. The values of phase angle are 45° to 90° for liquids, at *δ* = 45° crosslinking occurs, while below 45° to 0° *G*′ dominates over *G*″ indicating formation of solid materials. An ideal elastic deformation would result in *δ* = 0°, an ideal viscous deformation in *δ* = 90°. These characteristics can also be viewed from Figure 9. Namely, in the beginning, when formulations are in a liquid state, but their viscosity increase, the values of phase angle are between 90° and 45°. When crosslinking occurs for *G*′ = *G*″, the phase angle equals 45°. Then the decrease in the values of the phase angle towards 0° indicates the solid-state of the measured material. In this range, the epoxy formulations become highly crosslinked, molecules with larger and larger molar mass form, and their mobility is significantly decreased. The rigidity of the material increase, but during all stiffening processes also some reactive components may be present.

### 3.2. Characterstics of Protective Coatings

#### 3.2.1. Surface Properties

**Figure 10:**
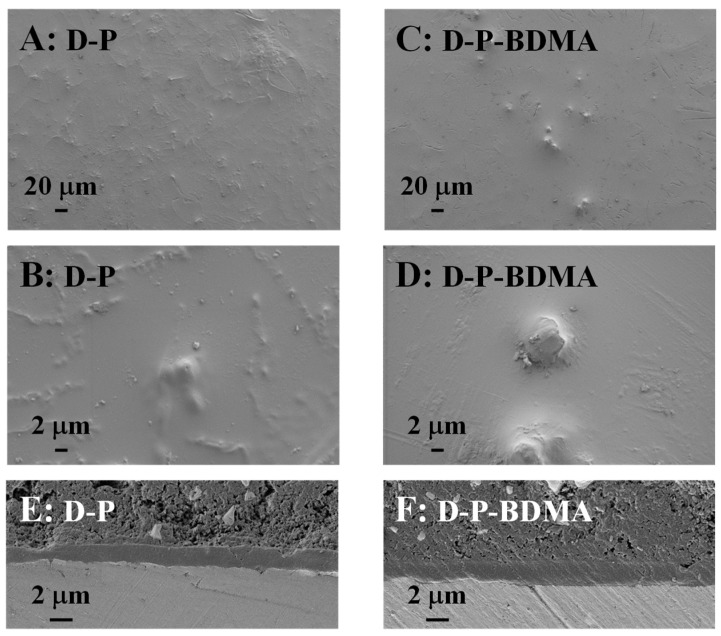
SEM micrographs of protective coatings at different magnifications: (**A**,**B**) D-P coating and (**C**,**D**) D-P-BDMA coatings. (**E**,**F**) Cross-cut SEM micrographs of D-P (**E**) and D-P-BDMA (**F**) coatings.

Protective efficiency of coatings is usually affected also by their surface morphology, originating from the intensity of the interface between the coating surface and the corrosive media. Consequently, SEM analyses of the surface and cross-cut of D-P and D-P-BDMA coatings are shown in Figure 10. The surface of the D-P coating (Figure 10A,B) reveals tiny inhomogeneities. On the contrary, the surface of the coating with the BDMA accelerator (Figure 10C,D) shows the presence of some elevated particles, but the surrounding surface is more homogeneous compared to the D-P coating. This tentatively suggests that when the accelerator was added the surface became more homogeneous. Nevertheless, some particle formation was observed also in this coating with the accelerator. The SEM of the cross-cut examples showed a compact coating structure for either coating without or with accelerator which is a prerequisite for sufficient protective efficiency (Figure 10E,F).

Besides, AFM images were recorded for D, D-P and D-P-accelerator coatings (Figure S4 in Supporting Information). The surface roughness is reported in Table 3. The AFM image of D coating shows areas with spherical particles in the coating composition with the size of a few hundreds of nanometers to the size of a micron. The measurements show random particle distribution. Moreover, similar morphology with some spherical particles can be observed in the D-P coating (Figure S4). Surface roughness values (*SR*), calculated from images demonstrate a considerably higher value for the coating composed of D compared to the D-P coating. 

As already shown by SEM (Figure 10), the addition of accelerators causes a more uniform and homogenous morphology of the coatings (Figure S4C–E in Supporting Information). As well, the addition of accelerators decreased the average roughness values (Table 3), ranging from 91 nm for the coating with BDMA to the smallest value of 58 nm obtained for the coating with Curezol (Figure S4C,E). Such a difference can originate from added accelerators. Specifically, all three accelerators stimulate the opening of the glycidoxy rings, which mitigated the reactions of crosslinking. Despite the reactions start at lower temperatures in the presence of accelerators, the support in ring-opening enabled more uniform crosslinking. Except in surface roughness, AFM images do not reveal any significant differences among accelerators (Figure S4C–E). The eventual bonding of Curezol or 2-MeIm in the epoxy coating does not influence their images. 

#### 3.2.2. Electrochemical Characteristics

**Figure 11:**
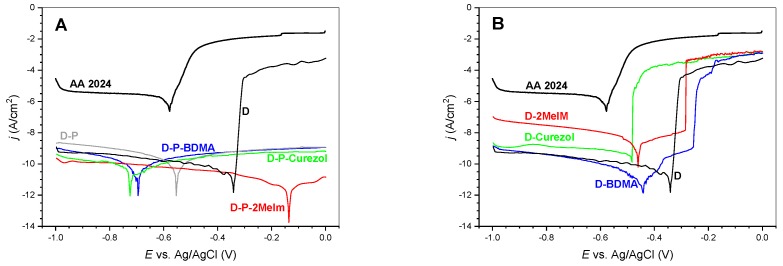
Potentiodynamic polarization curves of protective coatings; (**A**) D-P-based formulations and (**B**) D-based formulations.

Potentiodynamic polarization is a quick test of the protective efficiency of investigated coatings. The test is relative and the measurements should be performed in the same way and conditions. Figure 11 reports the potentiodynamic curves for D-P- and D-based coatings without and with accelerators in comparison. The difference in the current density between the measurement of the uncovered AA 2024 and the substrates covered with coatings reveals their protective efficiency. It is obvious that the shape of the potentiodynamic curves obtained for either of the D-P-based coatings shows its considerably improved performance with regard compared to D coating (Figure 11A). Even when either of accelerators was added into the D formulation (D-accelerator coatings in Figure 11B), the shape of the curves did not improve but resembled that of the D coating. Namely, the anodic current density for the D and D-accelerator coatings increased significantly with the increase in the potential. On the other hand, the current density of the D-P-based coatings remained low for all examined potentials. This showed the excellent protective efficiency of the D-P and D-P-accelerator coatings. Their compact structure (Figure 10E,F) prevents the formation of corrosion products at the interface coating substrate.

## 4. Summary and Conclusions

Epoxy coatings are known for their excellent protective efficiency in corrosion applications. Mostly, increased protective efficiency can be achieved through the formation of advanced epoxy nanocomposites [5,6,7,8]. The addition of suitable nanoparticles can result in improved temperature, chemical, and electrochemical resistance. An important parameter for the formation of nanocomposite materials is the appropriate incorporation of nanoparticles in the polymer matrix. Although in some cases reinforcement can be achieved by the simple addition of nanoparticles, their eventual bonding into the epoxy matrix can prevent leakage, migration, and agglomeration. Consequently, homoleptic octaglycidyl-POSS was used in this study. Specifically, 20 wt.% of DGEBA precursor was exchanged by GlyPOSS in reaction with DICY hardener. Three different accelerators that promote the opening of the glycidoxy rings were also tested.

DSC (Figure 3), temperature-dependent FT-IR absorbance (Figure 4, Figure 5, Figure 6 and Figure 7) and rheology (Figure 8) measurements confirmed that the addition of accelerators induced the lowering of the temperature at which the crosslinking processes occur. Such an effect is desired from the industrial viewpoint, significantly simplifying the production procedures and reducing the production costs. DSC (Figure 3) revealed a similar lowering of crosslinking temperature for 2-MeIm (131 °C) and Curezol (130 °C), while somewhat lower peak temperature was obtained by BDMA (136 °C). Temperature-dependent FT-IR absorbance measurements further differentiated among the accelerators. When isothermal treatment was performed at 150 °C (Figure 7A), the Curezol was the quickest in its action. BDMA was found the slowest during isothermal treatment at 120 °C (Figure 7B). Rheologically, the lower plateau values of elastic *G*′ and viscous *G*″ moduli for D-P-BDMA formulation showed that the BDMA accelerator, as it did not take part in crosslinking, led to less strong internal structure compared to 2-MeIm and Curezol (Figure 8). Temperatures at which *G*′ = *G*″ were similar for all three accelerators, although slightly lower (122 °C) when accelerator 2-MeIm was used. The described findings reflect the basic characteristics of the chosen accelerators. According to their structure, all three accelerators function as glycidoxy ring openers. However, Curezol can also contribute to crosslinking processes via primary amino groups on the triazine ring and 2-MeIm via aromatic secondary amine. These differences in the structures of accelerators reflect in the crosslinking behavior of the D-P-based formulations. All three applied measurement techniques differentiate BDMA accelerator from 2-MeIm and Curezol ones. The double function of the latter two, being openers for the glycidoxy rings and their possibility to collaborate in the crosslinking, prevented any final decision on the superior action of either of them. Interestingly, the rheological time sweep experiments performed to determine gel time (*G*′ = *G*″) based on viscoelastic parameters showed even its decrease when 1-benzyl-2-methylimidazole catalyst was added to DGEBA/triethylene-tetraamine formulation [29]. The *G*′ and *G*″ crossovers were determined isothermally at five different temperatures from 60 to 100 °C and only in case of some diluted formulations an accelerative effect of the catalyst was noted. This pointed to the importance of the presence of aromatic secondary amine in the structure of 2-MeIm (Figure 1). Anyhow, the slight differences in our DSC (Figure 3), FT-IR absorbance (Figure 4, Figure 5, Figure 6 and Figure 7) and rheology (Figure 8) measurements do not allow the decision on the preference. The preference may, however, be set via experimental demands. Namely, while the admixture of BDMA or 2-MeIm is straightforward, the addition of Curezol might occasionally result in the formation of an opaque formulation. This is a consequence of the low solubility of Curezol in organic solvents and water.

The surface roughness values obtained from AFM (Figure S4 in Supporting Information) showed distinct changes among the surfaces. When accelerators were not applied, i.e., neat D and D-P coatings, the surfaces revealed the presence of spherical particles and inhomogeneities in AFM images. The addition of either of accelerators resulted in lower values of the surface roughness (Table 3). SEM micrographs (Figure 10) confirmed the rougher surface of the D-P coating concerning the D-P-BDMA coating with the BDMA accelerator. It is worth mentioning, that also the coating with BDMA contained certain elevated areas, but the surrounding surface was much flatter and more homogeneous (Figure 10C,D). The SEM measurements of the cross-cut samples (Figure 10E,F) revealed the compact inner structures of D-P and D-P-BDMA coatings. Such a compact structure also resulted in the extremely good protective efficiency of D-P and D-P-accelerator coatings with regard to the neat D coating (Figure 11).

In conclusion, we can say that the time-dependent FT-IR absorbance measurements showed that partial exchange of DGEBA with GlyPOSS resulted in curing at a somewhat lower temperature. The reason probably lies in the eight glycidoxy groups that are positioned in the corners of this homoleptic cube-shaped GlyPOSS. The further lowering of the temperature of curing was achieved by the addition of various accelerators. It was found that the action of the accelerators considerably depends on their structure. Although all three accelerators are capable of the opening of glycidoxy rings, only Curezol and 2-MeIm can collaborate in crosslinking reactions. Specifically, Curezol can bind via two primary amino groups and 2-MeIm through aromatic secondary amine. Potentiodynamic polarization tests showed that all coatings comprising GlyPOSS show better protective efficiency compared to neat DGEBA/DICY (D) coatings. Consequently, our material is a promising candidate for a wide range of applications, such as coatings for food cans, white goods, etc.

## Figures and Tables

**Table 1:** Thermal properties of D-P-based formulations used for the preparation of coatings.

Sample	*T*_onset_^1^ (°C)	*T*_peak_^2^ (°C)	Δ*H* ^3^ (J/g)	*T*_g_^4^ (°C)
D-P	112	168	−506	116
D-P-BDMA	76	125	−345	128
D-P-2MeIm	62	131	−363	113
D-P-Curezol	78	130	−405	120

^1^*T*_onset_—onset temperature; ^2^*T*_peak_—temperature of the peak; ^3^ Δ*H*—enthalpy of the curing reactions; ^4^ *T*_g_—glass temperature.

**Table 2:** Rheological characterization of D-P-accelerator formulations used for the preparation of coatings.

Sample	*t*^1^ (min)	*T*^2^ (°C)	*T*_average_^3^ (°C)
D	66	155	
D-BDMA	50	123	
D-2MeIm	49	119	122
D-Curezol	51	125	
D-P	62	146	
D-P-BDMA	51	124	
D-P-2MeIm	50	122	124
D-P-Curezol	51	125	

^1^*t*—time, at which *G*′ = *G*″; ^2^ *T*—temperature, at which *G*′ = *G*″; ^3^ *T*_average_—average temperature, at which *G*′ = *G*″ for all three formulations with accelerators.

**Table 3:** Morphological and electrochemical characterization of the protective coatings.

Protective Coating	*d*^1^ (μm)	*SR*^2^ (nm)	*E*_corr_*^3^* (V)	*j*_corr_^4^ (A/cm^2^)	Protective Coating	*E*_corr_*^3^* (V)	*j*_corr_^4^ (A/cm^2^)
D	1.2	426	−0.336	1.8 × 10^−11^			
D-P	1.6	148	−0.549	2.8 × 10^−11^	D	−0.336	1.8 × 10^−11^
D-P-BDMA	1.5	91	−0.695	9.4 × 10^−12^	D-BDMA	−0.695	9.4 × 10^−12^
D-P-2MeIm	3.3	80	−0.138	2.6 × 10^−12^	D-2MeIm	−0.138	2.6 × 10^−12^
D-P-Curezol	3.5	58	−0.721	1.7 × 10^−11^	D-Curezol	−0.721	1.7 × 10^−11^

^1^*d*—thickness; ^2^*SR*—surface roughness; ^3^*E*_corr_—corrosion potential; ^4^*j*_corr_—corrosion current density.

## Supplementary Materials

The following are available online at https://www.mdpi.com/2073-4360/12/3/591/s1, Figure S1: FT-IR absorbance spectra of precursors DGEBA and GlyPOSS, and initial D and D-P formulations, Figure S2: Time-dependent FT-IR absorbance spectra during the thermal curing process of D-P formulation, Figure S3: Time-dependent FT-IR absorbance spectra during the thermal curing process of D formulation, Figure S4: AFM images of protective coatings: (A) D, (B) D-P, (C) D-P-BDMA, (D) D-P-2MeIm, (E) D-P-Curezol.
