# Supplementary material for: Tailored Crosslinking Process and Protective Efficiency of Epoxy Coatings Containing Glycidyl-POSS
Source: Polymers (Basel). 2020 Mar 5;12(3):591. doi: 10.3390/polym12030591 (PMC7182962; doi:10.3390/polym12030591)
Supplement: Supplementary file 1 [file polymers-12-00591-s001.pdf]

polymers

MDPI

Supporting information

Tailored crosslinking process and protective efficiency of epoxy coatings containing glycidyl-POSS

Mirjana Rodošek, Mohor Mihelčič, Marija Čolović, Ervin Šest, Matic Šobak, Ivan Jerman and Angelja K. Surca

---

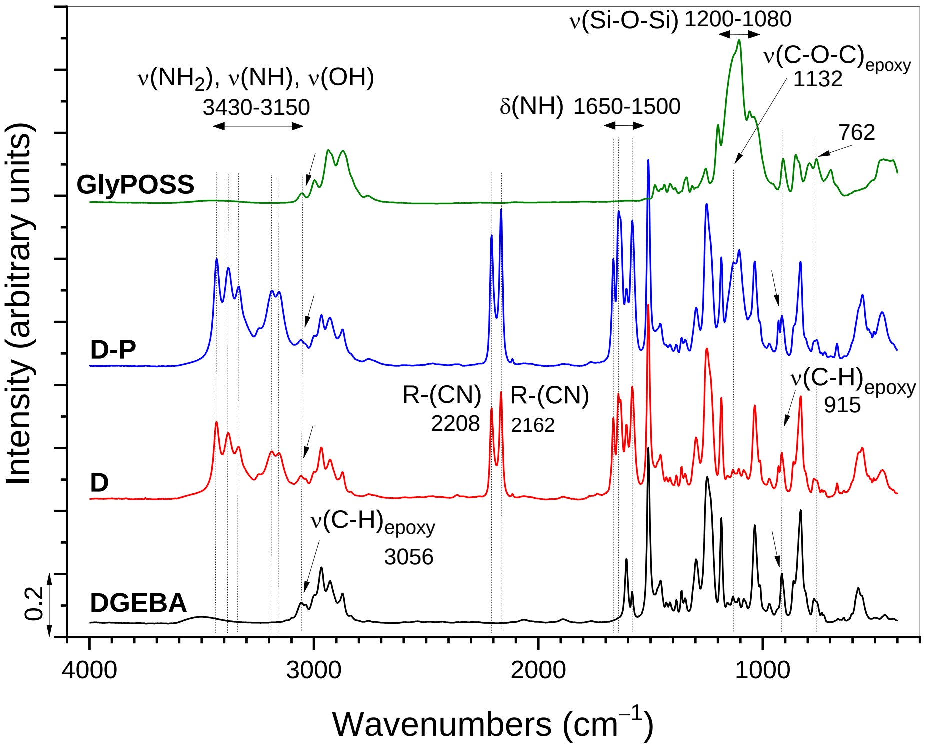

Figure S1. FT-IR absorbance spectra of precursors DGEBA and GlyPOSS, and initial D and D-P formulations.

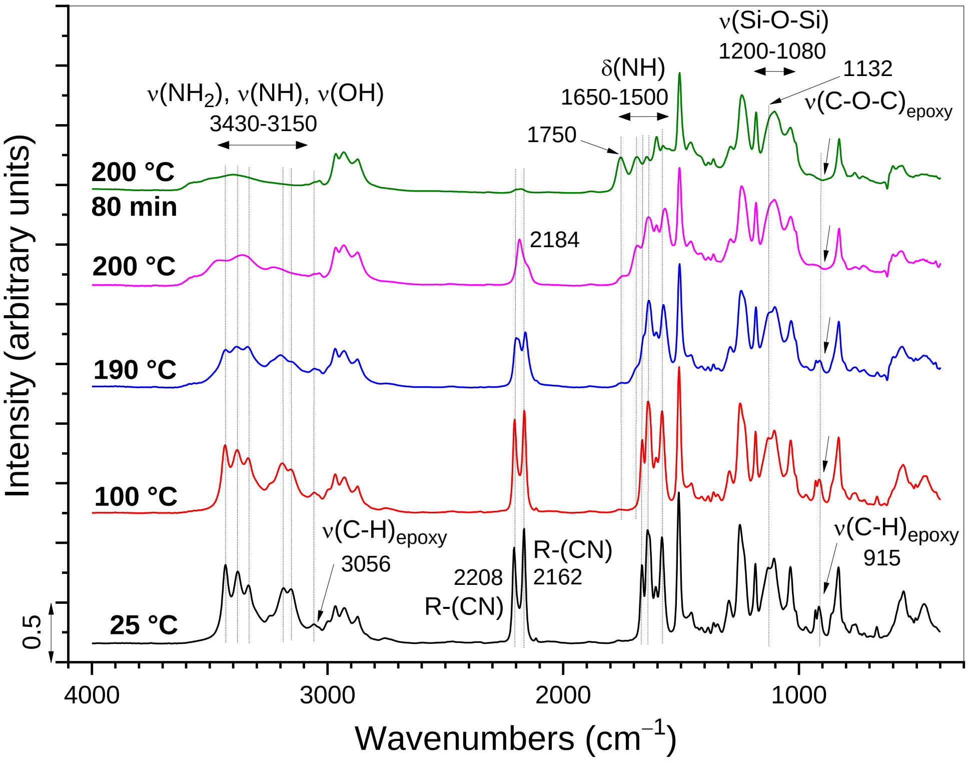

Figure S2. Time-dependent FT-IR absorbance spectra during the thermal curing process of D-P formulation.

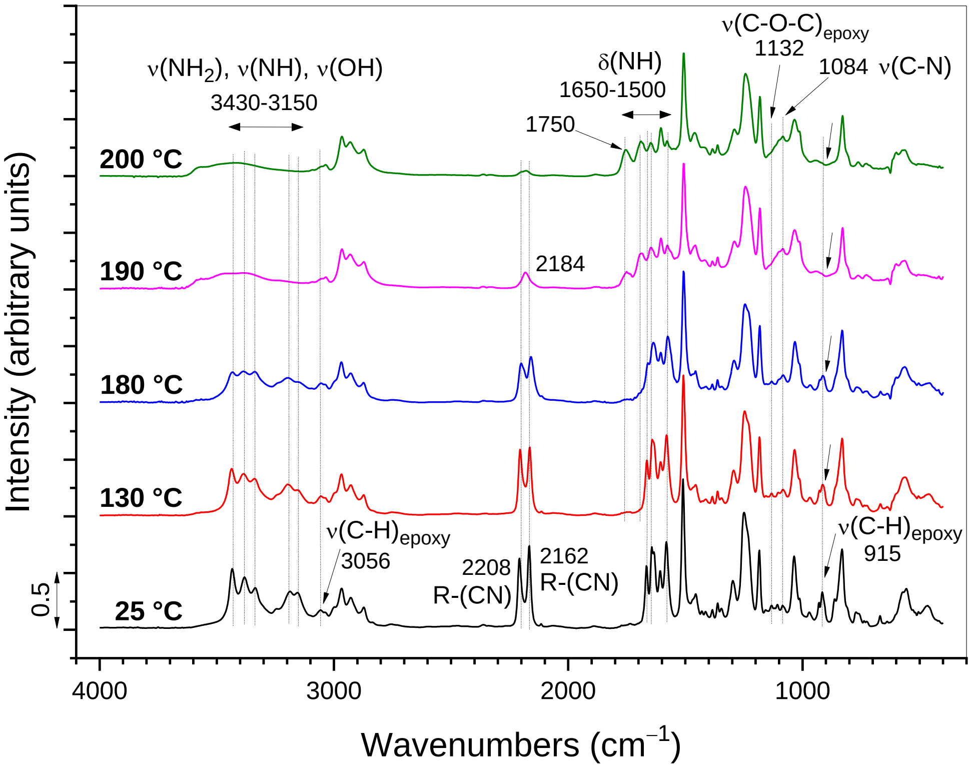

Figure S3. Time-dependent FT-IR absorbance spectra during the thermal curing process of D formulation.

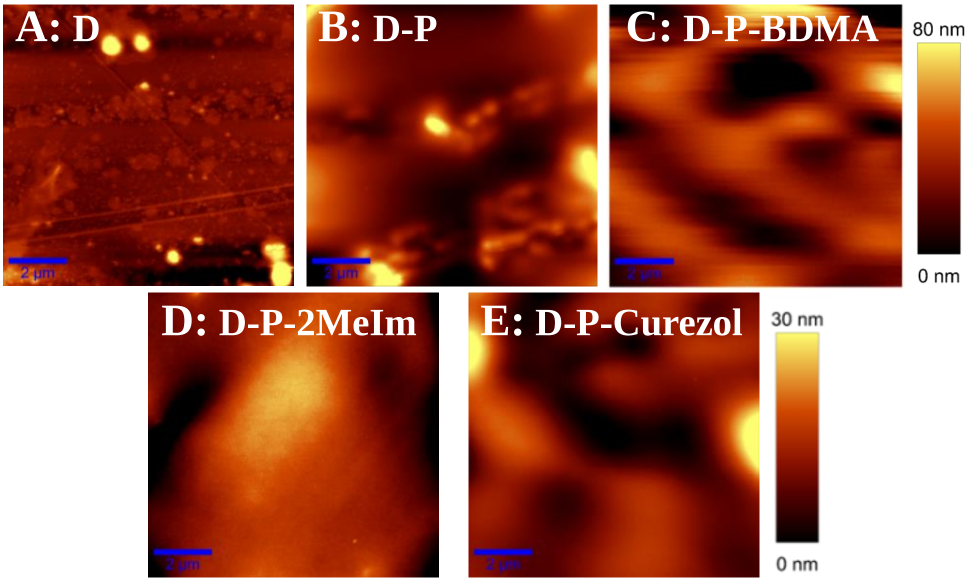

Figure S4. AFM images of protective coatings: A) D, B) D-P, C) D-P-BDMA, D) D-P-2MeIm, E) D-P-Curezol.
